# Inhibition and Eradication of *Pseudomonas aeruginosa* Biofilms by Host Defence Peptides

**DOI:** 10.1038/s41598-018-28842-8

**Published:** 2018-07-11

**Authors:** Hongwei Chen, Richard W. Wubbolts, Henk P. Haagsman, Edwin J. A. Veldhuizen

**Affiliations:** 1 College of Animal Science, Rongchang Campus, Southwest University, Chongqing, 402460 China; 2 Department of Infectious Diseases & Immunology, Faculty of Veterinary Medicine, Utrecht University, Utrecht, The Netherlands; 3 Department of Biochemistry and Cell Biology, Faculty of Veterinary Medicine, Utrecht University, Utrecht, The Netherlands

## Abstract

*P*. *aeruginosa* is a notorious biofilm producer that causes a wide variety of acute and chronic infections. In this study the *in vitro* anti-biofilm activity of 13 Host Defence Peptides from different species was tested against *P*. *aeruginosa* biofilms. Most HDPs were able to prevent biofilm attachment, due to their antimicrobial effect on planktonic bacteria in the starting inoculum. Activity of HDPs against pre-formed biofilms was also observed, although mainly at short incubation times. Several HDPs were able to kill bacteria in the biofilm (colony counting of biofilm associated bacteria) but only CRAMP eradicated the whole biofilm (crystal violet staining). These results were quantitatively confirmed by confocal microscopy studies using a live/dead stain of the biofilms. Furthermore, for chicken CATH-2 (one of the more potent HDPs) it was shown that the peptide could indeed penetrate the biofilm structures and kill bacteria within the biofilm. These studies highlight the potency but also the limitations of HDPs as new potential anti-biofilm agents.

## Introduction

Biofilms are three-dimensional bacterial communities attached to surfaces, either living or abiotic, surrounded by an extracellular matrix consisting of bacterium-derived DNA, proteins and exopolysaccharides^1–3^. In nature, bacteria are considered to be mainly found in the form of biofilms, and it is estimated that as little as 0.1% of the total microbial biomass is actually in the planktonic mode of growth^4^.

Biofilm development is initiated by bacteria associating to a surface forming microcolonies. Subsequently, these develop into mature biofilms, dependent on several factors including bacterial cell density and availability of nutrients. However, the complex pathways leading to full development of biofilms, are not completely understood but involve quorum sensing affecting numerous complex regulatory gene networks^5^. The formation of a biofilm is considered a survival strategy to environmental stress such as pH, UV damage, hydrogen peroxide and metal toxicity but also towards the human immune response towards bacterial infection, including phagocytosis^5,6^. One bacterium that is particularly notorious for being able to form biofilms is *P*. *aeruginosa*. It can form biofilms in many environments and can cause a wide variety of chronic infections^7^. In addition, it is a major nosocomial pathogen in patients with cystic fibrosis (CF)^8,9^. The clinical relevance and the relative ease of biofilm growth has made this bacterium a model organism with respect to research on biofilm formation^10^.

There is a growing realization that many chronic infections are biofilm related. In fact, the NIH acknowledged that likely 80 percent of medical bacterial infections treated by physicians in the developed world are caused by organisms growing in biofilms^11^. However, most research characterizing the physiology of bacteria, has been performed on planktonic bacteria in liquid media. Similarly, discovery of all current antibiotics and other antimicrobial products are also based on activity towards planktonic bacteria^7^. Subsequently, established biofilms can be up to 1,000-fold more resistant to antibiotic treatment than planktonic bacteria, making them very resistant to current treatments^12^. This is partially because most antimicrobials are mainly effective against rapidly growing cells. However, several other causes can be identified including that the exopolymer matrix of the biofilm can restrict the diffusion of substances and bind antibiotics and components of the host’s immune system^12,13^.

The mismatch between current antibiotics and anti-biofilm activity has led to a rethinking in the optimal strategy to fight chronic biofilm related infections and an intensified search for new antimicrobials. One class of such new anti-biofilm compounds could be formed by Host Defence Peptides (HDPs). These peptides, originally termed antimicrobial peptides, are evolutionarily conserved molecules that serve in host defence against insults including microbial infections and inflammation^14^. In recent years, there has been an increasing appreciation for their anti-biofilm activities. For example, the human cathelicidin LL-37 was shown to inhibit biofilm formation of *P*. *aeruginosa* at 0.5 μg/ml, and disperse preformed biofilms at 4 μg/ml, far below its MIC value against planktonic bacteria under physiological conditions^10^. However, there is still only a limited number of articles about eradicating preformed biofilms^10,14–16^, especially considering the large number of naturally occurring HDPs.

Here, we report results from studies designed to determine activities of naturally occurring cathelicidins and the synthetically developed HDP-based peptide IDR1018 against *P*. *aeruginosa* biofilms. Several HDPs significantly reduced viability of biofilm-associated bacteria of established biofilms, while one HDP (CRAMP) was also able to efficiently eradicate the whole biofilm.

## Results

### Biofilm attachment assay

**Figure 1:**
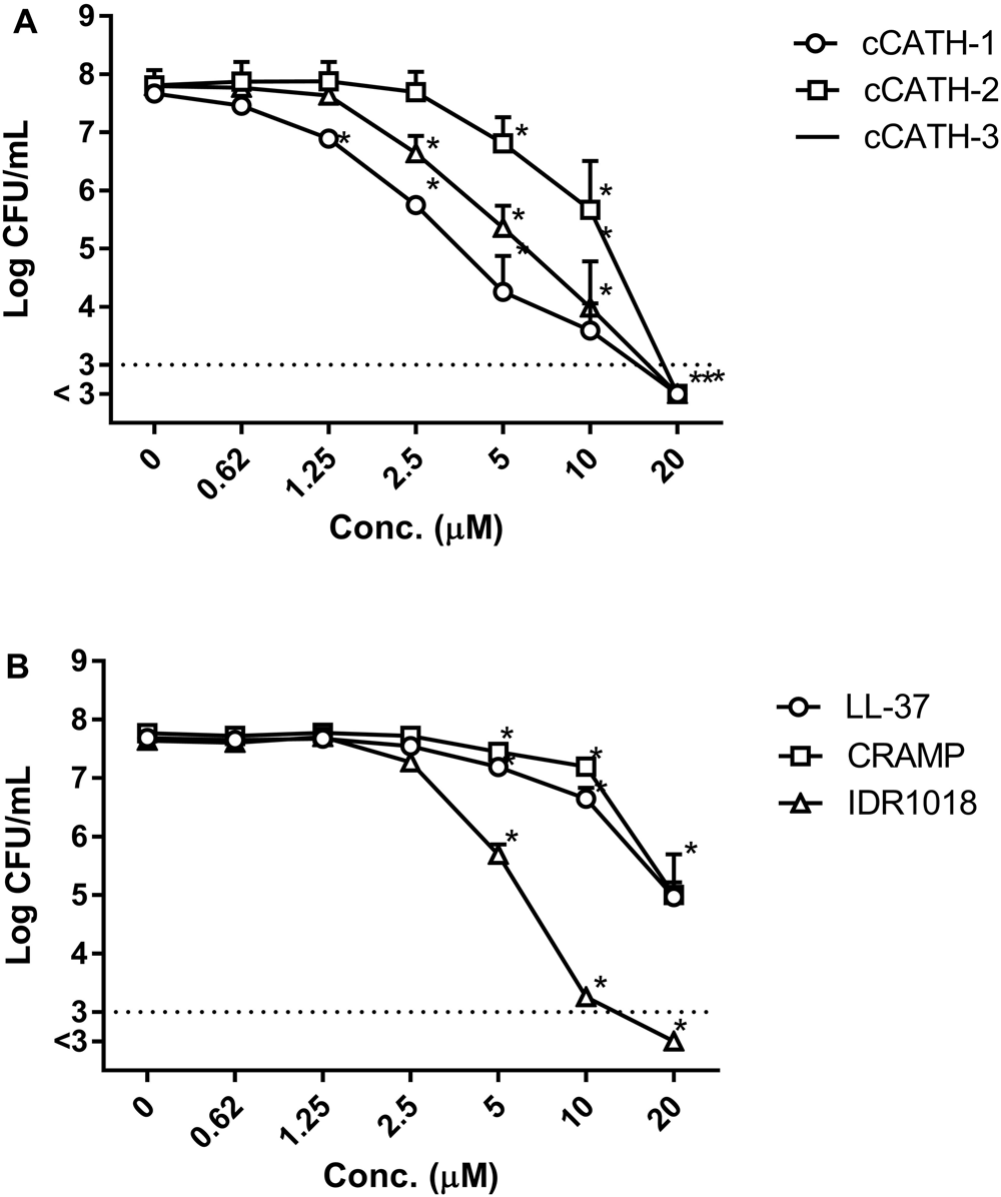
HDPs are active against planktonic bacteria. After 30 min of biofilm formation in the presence or absence of peptides, 5 μl of the medium was diluted and spread on TSA plates to determine viability of planktonic bacteria. (**A**) cCATH-1, 2 and 3, and (**B**) LL-37, CRAMP and IDR1018. Shown are averages of at least 3 independent experiments ± s.e.m. *Indicates statistical significance compared to the non-peptide control.

**Figure 2:**
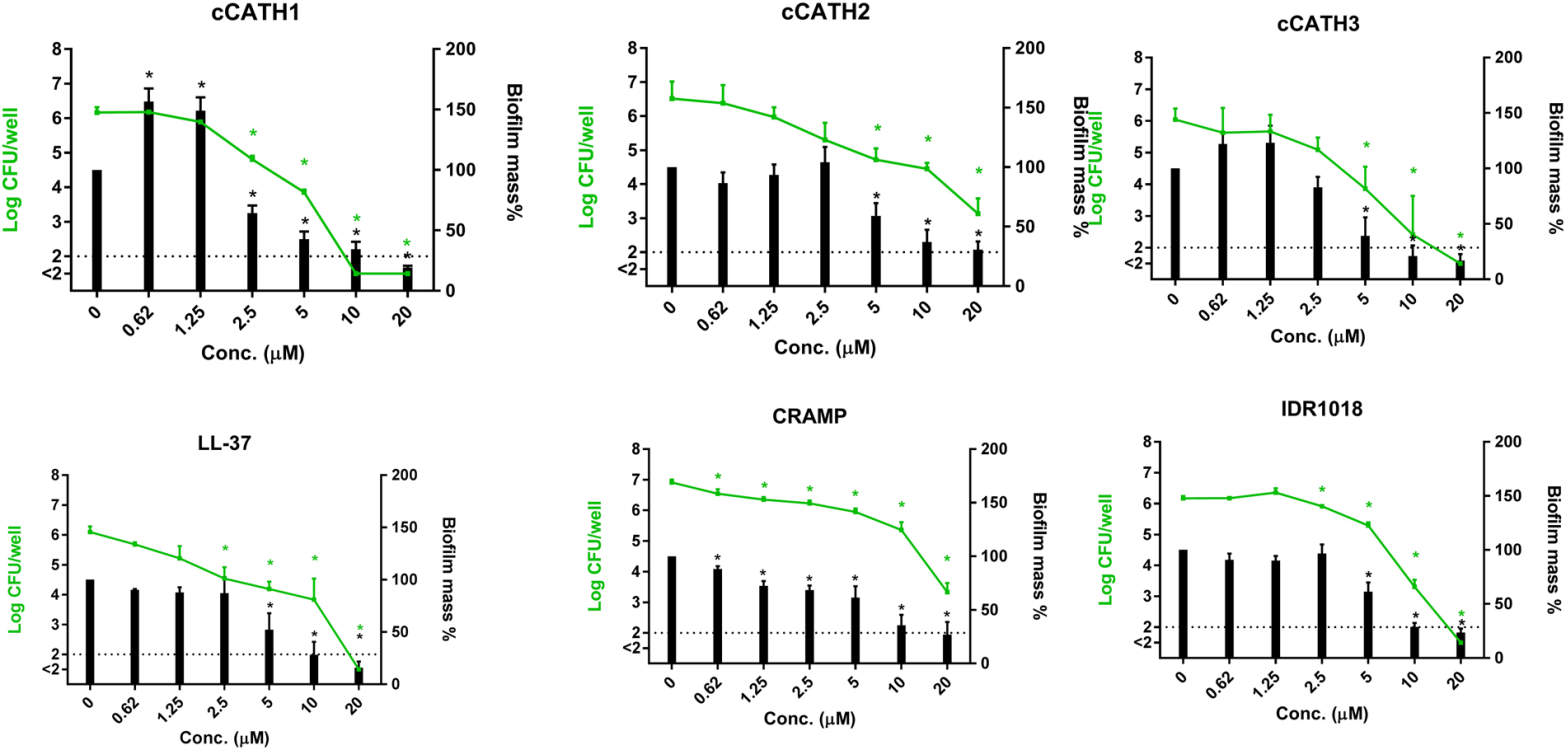
HDPs reduce biofilm attachment. Surface attachment of *P*. *aeruginosa* was allowed for 4 h at 37 °C as start of biofilm formation, in wells containing 0–20 μM cCATH1, 2 and 3, LL-37, CRAMP or IDR1018. Bars represent biofilm mass as determined by CV, while symbols and lines indicate the number of viable bacteria in the biofilm. Shown are averages of at least 3 independent experiments ± s.e.m. *Indicates statistical significance compared to the non-peptide control.

As a first step in determining anti-biofilm activity of HDPs, 3 peptides with described antibiofilm activity (LL-37, CRAMP and IDR1018) and 3 HDPs with described activity against planktonic *P. aeruginosa* (cCATH-1, 2 and 3), were tested for their ability to prevent biofilm formation. For this, *P*. *aeruginosa* was incubated with 0–20 μM peptide for 4 h (defined as the attachment phase). However, after 30 min (before biofilm formation properly started) a small aliquot of the medium was taken, diluted and plated on TSA plates to determine direct antimicrobial effects of the tested peptides against planktonic *P*. *aeruginosa* in the inoculum. As shown in Fig. 1, all tested peptides actually had a significant effect on viability of planktonic bacteria in the solution with several peptides reducing the number of bacteria more than 3 LOG (below the detection limit of 1000 CFU/ml) at 20 μM. Next, biofilm attachment was assessed in the presence of HDPs (Fig. 2). A detectable biofilm was formed after 4 h, in the absence of peptide, with approximately 2 × 10^6^ colony forming units (CFU) in a starting biofilm. With increasing amounts of HDPs, there was a clear reduction in number of viable bacteria and biomass of the biofilm. Clearly, the pattern of prevention of biofilm attachment, with effects starting at >10 μM, correlates to the direct antimicrobial activity of the peptides observed in Fig. 1, indicating that it is likely the initial reduction in planktonic bacteria that reduces the attachment of biofilm in the first 4 h. Interestingly, for cCATH-1 addition of relatively low amounts of peptide (0.62 and 1.25 μM) significantly increased the biofilm mass as detected by crystal violet staining.

### Inhibition of biofilm formation

The presence of HDPs during the full 24 h formation of biofilms showed only minor effects on the amount of biofilm formed after 24 h (Fig. S1). Only 20 μM cCATH-1 showed a significant reduction in the number of viable bacteria in the biofilm after 24 h. With respect to biofilm mass, no significant effects were observed and even a small tendency to higher biofilm mass was observed for some peptides. Thus, in general these results mainly show that although the initial biofilm attachment is affected by killing of planktonic bacteria, this difference disappears when biofilms have longer time to form (and bacteria have longer time to multiply).

### Activity of peptides against preformed biofilms

Preformed biofilms of *P*. *aeruginosa* were grown for 24 h at 37 °C in 96 wells plates, and then treated with peptides (0–20 μM). Optimization of incubation time (1–24 h) of preformed biofilms with HDPs was performed for cCATH-2, which indicated that effects on the number of viable bacteria in the biofilm were mainly observed after 1 h (Table S1). No effects were observed on biofilm mass at any time point. After that initial effect on viability, surviving bacteria in the biofilm could regrow again (as observed in the biofilm formation assays). Therefore, HDPs were further tested on preformed biofilms using 1 h incubation times.

### Activity of HDPs against preformed biofilms

**Figure 3:**
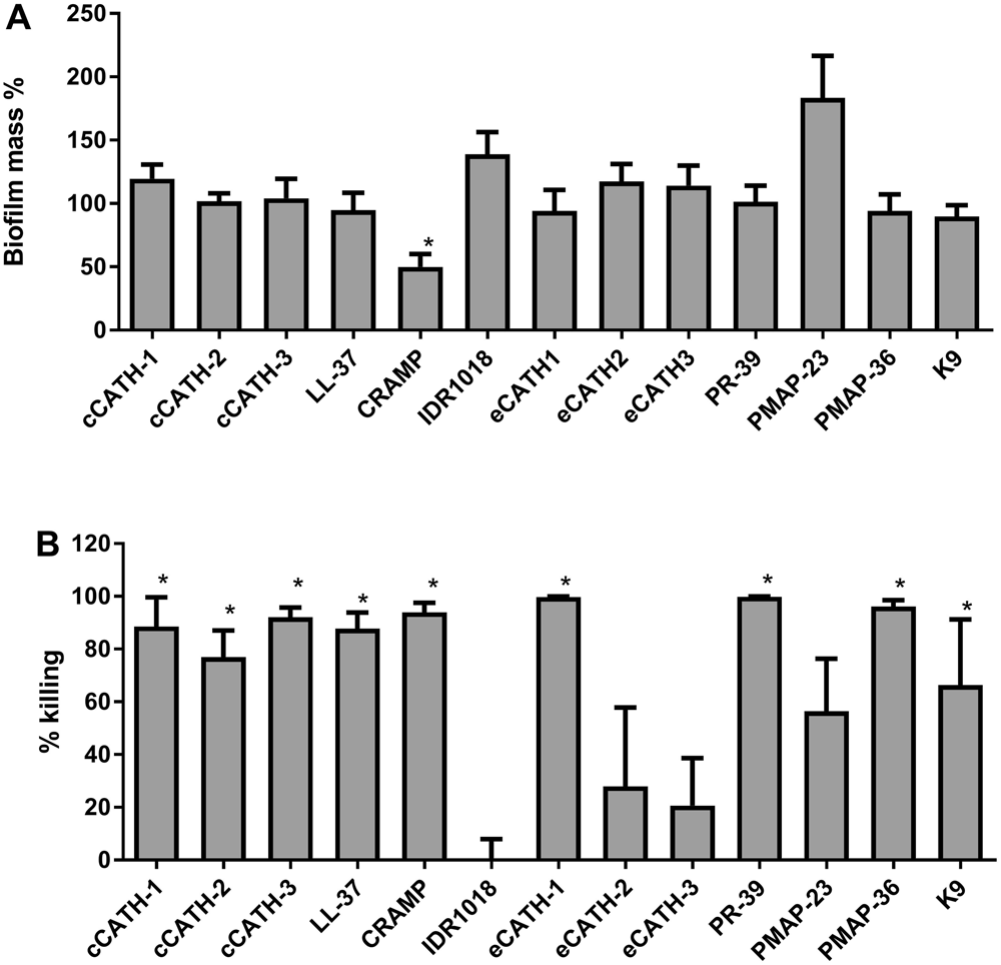
Activity of HDPs against preformed *P*. *aeruginosa* biofilm. *P*. *aeruginosa* biofilms were formed for 24 h at 37 °C, and subsequently treated with indicated peptides at 0–20 μM for 1 h. Viability and biofilm mass were determined using colony counting after solubilization of the biofilm, and CV staining respectively. For clarity only the data of 20 μM are shown. The complete data set with all concentrations of HDPs is shown in Fig. S2. Shown are averages of at least 3 independent experiments ± s.e.m. *Indicates statistical significance compared to the non-peptide control.

A broad set of 13 HDPs was tested against preformed *P*. *aeruginosa* biofilms. Several HDPs showed a dose-dependent reduction of the number of viable bacteria in the biofilm (Fig. S2). However, only marginal effects were observed on biofilm mass, with the exception of CRAMP. In Fig. 3, an overview is given for the large amount of data in Fig. S2, showing only the effects at 20 μM. Of all tested peptides, indeed only CRAMP was able to significantly reduce the biofilm mass by 50% (Fig. 3A). However, viability of bacteria in the biofilm was affected by many more HDPs (Fig. 3B). Except for PMAP-23, eCATH-2 and 3 and, surprisingly, IDR1018, all peptides caused significant killing (50–99%) of bacteria in the biofilm.

### CLSM observation HDP-treated biofilms

**Figure 4:**
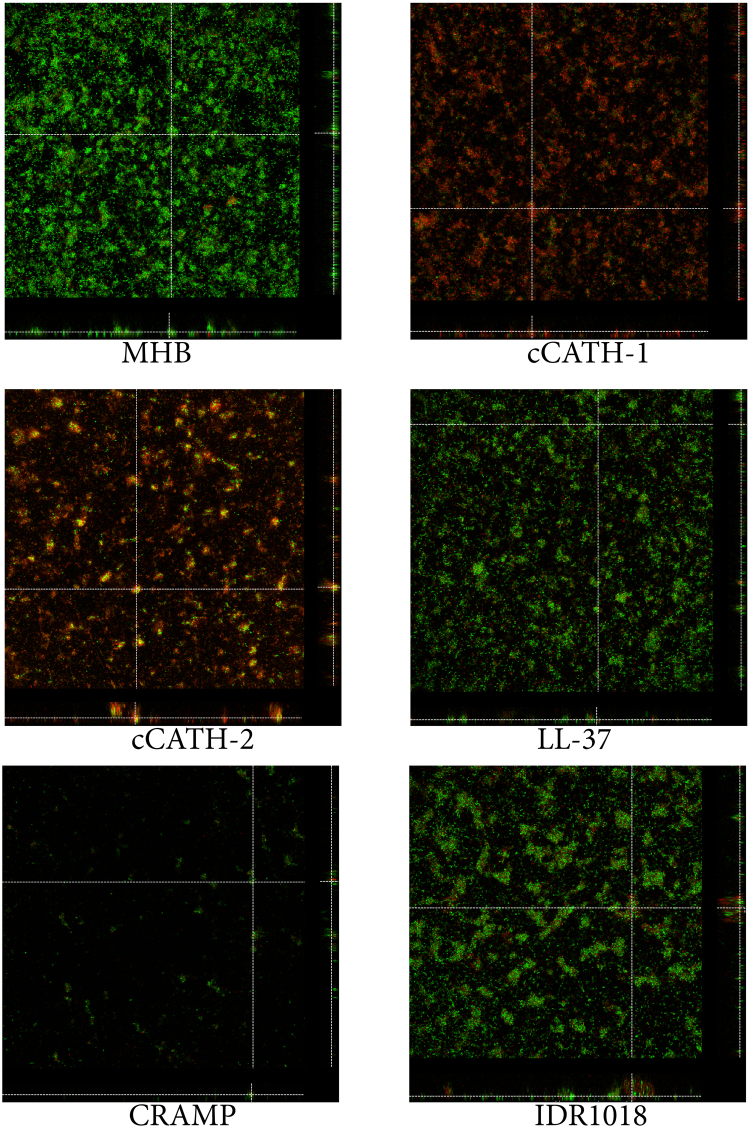
CLSM of HDP-treated *P*. *aeruginosa* biofilms. *P*. *aeruginosa* biofilms were formed for 48 h at 37 °C on chambered coverglass slides. After washing, biofilms were treated with HDPs (20 μM) for 1 h at 37 °C as described and subsequently stained with SYTO 9 and PI for 20 min in the dark. Images were acquired by CLSM using an ACS APO 20.0 × 1.30 oil objective lens. Representative orthogonal views of Z-stacks of HDP-treated biofilms are shown.

In order to gain further insight in the anti-biofilm activity of HDPs, confocal laser scanning microscopy (CLSM) was used to visualize preformed biofilms after treatment with selected HDPs. Biofilms were grown for 48 h instead of 24 h, since initial experiments indicated that on the different carrier (required for microscopy), biofilm formation took longer and also required gentle shaking. Biofilms were then treated for 1 h with peptide and stained with SYTO9 (live) and PI (dead) bacteria. When biofilms were incubated with MHB without peptide, an overall green staining was observed indicative of biofilms with mainly viable bacteria (Fig. 4). Biofilms consisted of a relatively thin layer with extrusions on top indicative of biofilm formation. When biofilms were treated with cCATH-1 and cCATH-2 mostly red stained, dead bacteria were observed. Similarly, LL-37 increased the number of dead bacteria in the biofilm, while CRAMP eradicated large parts of the biofilm. No large effects were seen when the biofilm was treated with IDR1018.

**Figure 5:**
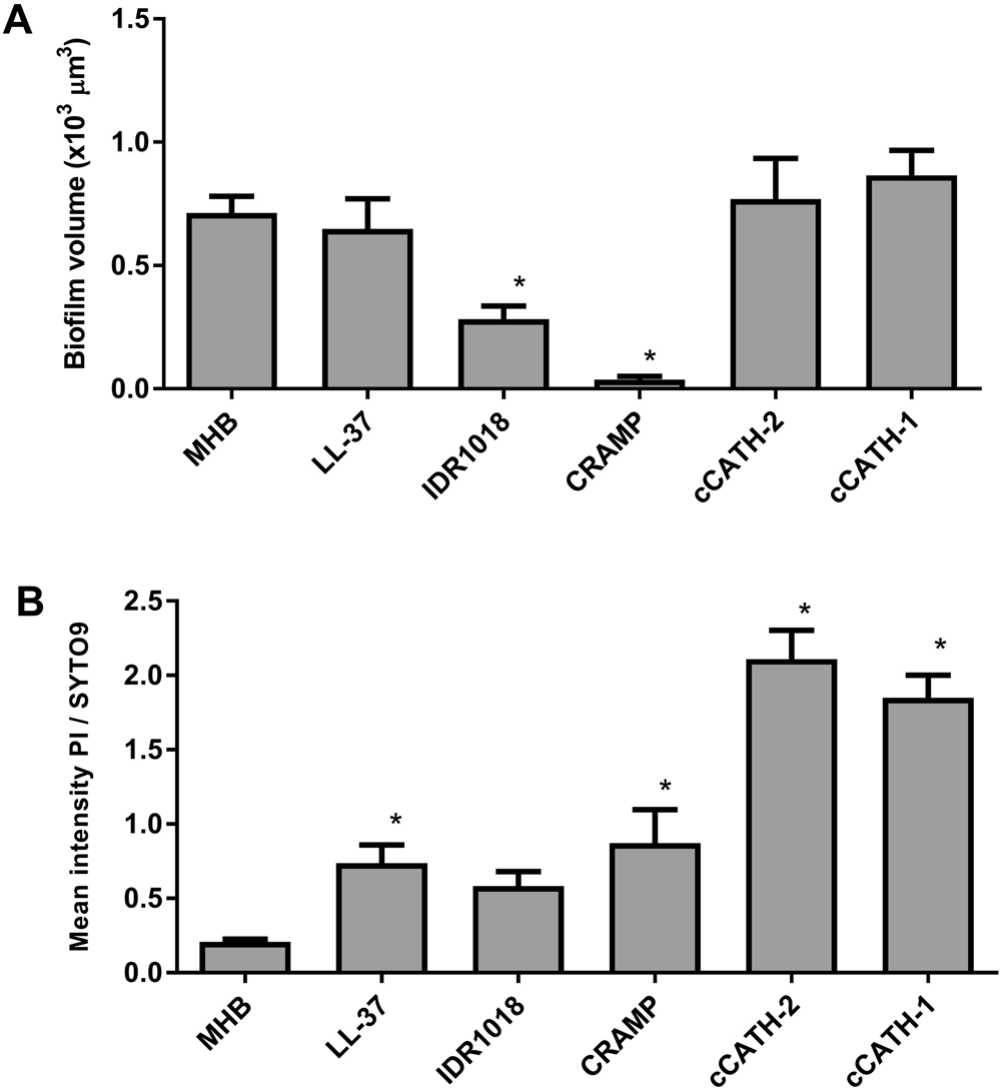
Quantitative analysis of biofilm volume and live/dead staining. Biofilms were formed and treated as described in Fig. 4. Biofilm protrusions were identified and total volume and staining of these protrusions was quantified. (**A**) Volume of HDP-treated biofilms. (**B**) PI/SYTO9 staining of bacteria in the biofilm. Of each biofilm a minimum of 4 representative images were obtained and each experiment was repeated ≥4 times. Shown are averages ± s.e.m. *indicates statistical significance compared to the non-peptide control.

After the qualitative determination, a quantitative assessment was made for the effect of HDPs on preformed biofilms. For this, of each biofilm, 4 representative images were taken for analysis and each experiment was performed ≥4 times. Of each biofilm, the thin layer of bacteria was deducted from the image, leaving only the biofilm protrusions for analysis. The total volume of protrusions (number of voxels in the 3D image) was determined as a measure of biofilm volume. CRAMP treatment resulted in a more than 19-fold reduction compared to the non-treated control (Fig. 5A). Surprisingly, also IDR-1018 showed a smaller but significant 50% reduction in total biofilm volume, but all other HDPs had no effect on biofilm volume. Subsequently, the intensity of the green and red stain inside biofilm protrusions was measured, indicative of bacterial killing in the biofilm (Fig. 5B). All peptides, except for IDR1018, increased the red/green ratio significantly compared to the untreated control. One should keep in mind that the ratio should not be interpreted as number of dead vs. live bacteria but is based on total fluorescence intensity of both dyes. Overall, the results correspond well with the earlier CV and viability assays on treated preformed biofilms in 96-well plates (Fig. 3).

**Figure 6:**
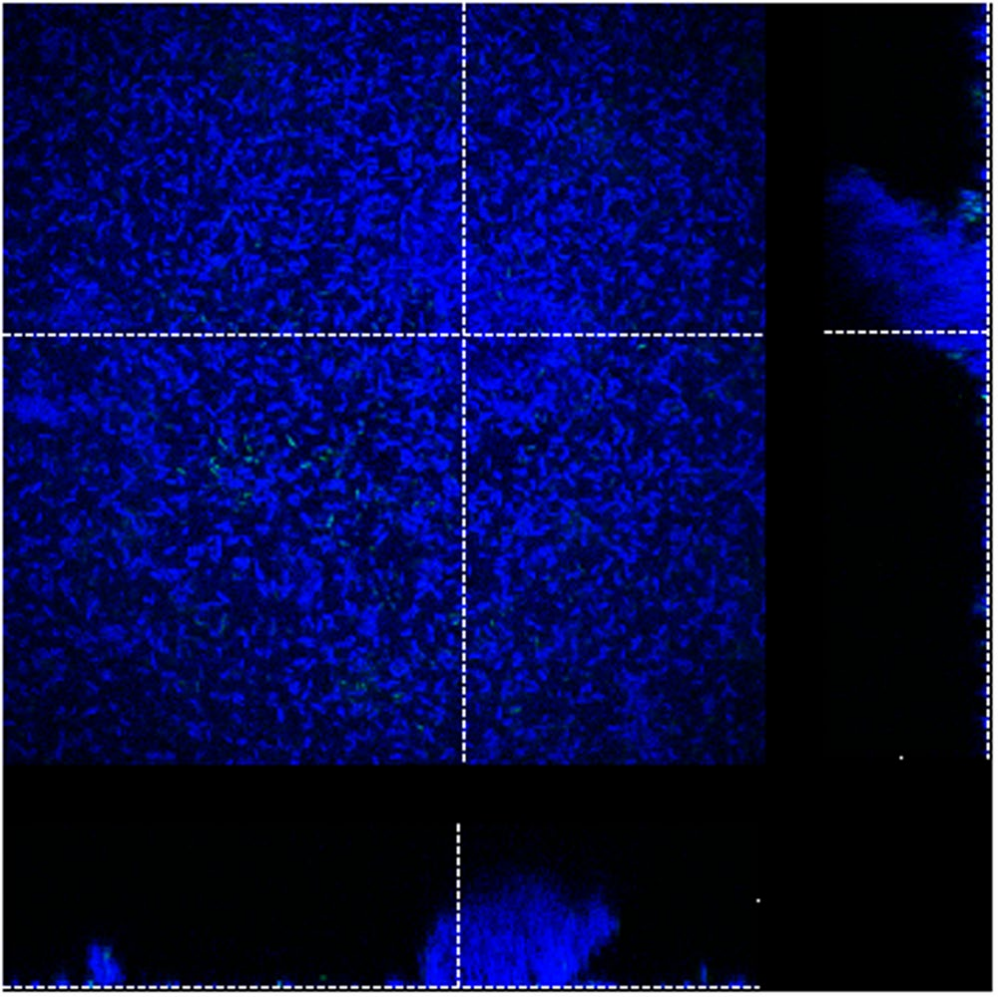
CY5-cCATH-2 penetrates a *P*. *aeruginosa* biofilm. Representative image of a *P*. *aeruginosa* biofilms treated with 20 μM CY5-cCATH2 for 1 h at 37 °C. After treatment, the biofilm was stained with SYTO 9 (green). Bound CY5-cCATH2 appears blue. Images were acquired by CLSM using an ACS APO 63.0 × 1.30 oil objective lens. A representative orthogonal view of Z-stacks of CY5-cCATH-2-treated biofilms is shown.

Finally, in order to obtain an indication whether the peptide could penetrate biofilms, a fluorescently (CY5) labelled version of cCATH-2 was used. Biofilms were treated with CY5-cCATH-2 for 1 h and the peptide localization was determined within the preformed biofilm (Fig. 6). Clearly CY5-cCATH-2 was able to penetrate the biofilm structures and (presumably) kill bacteria within the biofilm showing that the extracellular matrix was not (fully) blocking access of the peptide.

## Discussion

Treatment of bacterial infections, especially of the so-called ESKAPE pathogens, is becoming increasingly difficult, due to their increased resistance to conventional antibiotics. In addition, their susceptibility towards antibiotics is decreased dramatically when they are present in biofilms. Since over 65% of human infections are thought to be correlated to biofilm associated pathogens^17,18^, a need for new antimicrobials that are active, or even specific for prevention or treatment of bacterial biofilms is desperately needed.

Recently, host defence peptides or derivatives thereof, have demonstrated anti-biofilm activity^7,10,19–21^. HDPs were shown to be active both in prevention of biofilm formation and eradication of preformed biofilms. In this study we investigated the activity of 12 naturally occurring cathelicidins and a HDP derived peptide (IDR1018) with described anti-biofilm activity against *P*. *aeruginosa* biofilms *in vitro* and *in vivo*^14,22,23^.

With respect to the inhibition of biofilm formation, our results indicated that several peptides were capable of decreasing the initial attachment (4 h) of bacteria to the surface material. However, this was very likely due to killing of planktonic bacteria in the inoculum by the peptides’ direct antimicrobial activity. Obviously, even at a concentration below the MIC of the peptide (often referred to as sub-MIC), considerable killing of bacteria is achieved by HDPs and this should not be interpreted as specific anti-biofilm activity. In addition, in biofilms formed and treated with HDPs in flow systems, one should be aware that local concentrations of HDPs could be higher than the initial concentration in solution due to binding of peptide to the biofilm leading to potential MIC- or higher local concentrations^24^, again emphasizing that sub-MIC in relation to specific anti-biofilm (versus general antibacterial activity) should be used cautiously.

Despite the initial killing/reduction in biofilm attachment, the long-term effect of peptides was relatively low in our experimental set-up. Apparently, surviving bacteria were able to outgrow and form a biofilm with comparable mass after 24 h compared to control, non-treated bacteria. Basically, partial killing of the initial inoculum results in a delay of biofilm formation but apparently not in a lasting reduction. This result is different from several studies where HDPs were observed to have long lasting (24 h) effects, including LL-37, a peptide present in our tested panel^7,25^. On the contrary, our results are completely in line with earlier experiments in our group where we observed rapid, unhindered regrowth of planktonic bacterial populations after sub-MIC partial killing (up to 99%) of bacteria within 2–3 hours after peptide treatment^26,27^. This is an indication that during the initial killing process, HDPs are either inactivated by proteases, attached to bacterial remnants or in some other way unavailable for killing newly grown bacteria.

Activity of HDPs against preformed biofilms was tested on biofilms grown in the standard conditions (plastic 96-well plates) using CV staining and viability testing, and on biofilms grown on glass using confocal microscopy to determine HDP’s activity. In our microscopy setup, visualization of biofilms on a glass surface showed that these biofilms on glass consisted of a relatively thin layer of only 1–2 bacteria with protrusions on top which were considered to resemble ‘true‘ biofilms. Similar biofilms on top of a ‘monolayer’ were observed in other studies^28,29^, although there are also examples of thicker, more homogenous biofilms, depending on bacterial strain and growth conditions (flow systems)^10,25,30^. In our newly setup semi-quantitative analysis of anti-biofilm activity we only included bacteria in the protrusions of the biofilm to obtain a better representation of the peptide’s anti-biofilm activities. Interestingly, this new analysis corresponded very well with the classic CV and viability assays on the polystyrene-grown biofilms. Several peptides at a 20 μM concentration were able to kill bacteria within the biofilm. The overall pattern of activity of HDPs against preformed biofilms corresponds well to the activity of these HDPs against planktonic bacteria in broth dilution assays (Table S2). HDPs with a low MIC and MBC value, generally have higher activity against preformed biofilms as well, with the exeption of CRAMP which is not a strong antimicrobial against planktonic *P*. *aeruginosa*. For cCATH-2 we also showed that the (labeled) peptide can actually penetrate and kill bacteria inside the biofilm. However, none of the peptides, with the exception of CRAMP and to a lower extent IDR1018 were able to eradicate the physical biofilm. It is unclear whether this indicates that these biofilm-eradicating peptides have a different interaction with the EPS of the biofilm, or alternatively have a different killing mechanism of bacteria inside the biofilm, affecting the EPS and leading to release of the biofilm from the surface. Future experiments aimed to investigate the mechanisms of anti-biofilm activity of especially these eradicating peptides would be very useful to obtain an indication of the potential of HDPs as anti-biofilm agents.

Despite the increasing number of studies on the anti-biofilm activity of HDP or related peptides, there is not much known about mechanisms of activity. Since HDPs do not rely on growth phase or metabolic activity of bacteria, it is possible that the main mechanism involved in HDP’s anti-biofilm activity is similar to their activity towards planktonic cells. Much more is known about these latter mechanisms which for many HDPs seems based on bacterial membrane perturbations by these amphipathic peptides^31,32^. However, several HDPs have also been shown to target intracellular targets such as proline-rich peptides^33,34^ and IDR peptides^22^ as well as for cCATH-2 which traverses the bacterial membrane^26^. There are some indications though for specific anti-biofilm activity of HDPs. For LL-37, it was observed that as many as 786 genes were differentially regulated after 4 days of biofilm growth in the presence of the peptide. Genes related to quorum sensing and flagella assembly were downregulated, while genes related to type IV pili biogenesis and function were upregulated^10^. However, it is impossible to determine whether these are directly or indirectly influenced by LL-37 after such a long incubation period. Most work on the exact working mechanism of anti-biofilm activity has been performed on IDR1018. This peptide was mildly active against planktonic *P*. *aeruginosa* (MIC = 16–41 μM, partially depending on the growth medium)^22,35^ but specifically prevented biofilm formation, and was able to eradicate existing biofilms. IDR1018 supposedly interacts with two bacterial signaling nucleotides: guanoside 5’diphosphate 3 diphosphate (ppGpp) and the 5′triphosphate (pppGpp) version. These conserved molecules are induced in stress conditions and play a role in biofilm formation. A similar working mechanism involving (p)ppGpp degradation was later described by the same group for a set of new designer peptides loosely based on IDR1018, with sometimes even higher activity^36^. However, a follow-up study strongly challenged the observation that (p)ppGpp is a specific target of IDR1018^24^ and alternative/additional mechanisms should be considered.

Our current studies are useful as a first indication about antibiofilm activities of HDPs but there are clear limitations present. Susceptibility of biofilms could significantly be different from naturally occurring biofilms of clinical (more virulent) isolates of *P*. *aeruginosa* or from multispecies biofilms. In addition, the activity of HDPs seems to be present at relatively high concentrations, close to concentration where toxicity is observed for some of the HDPs, and for only limited time periods (Table S1, Fig. S2). This makes the use of HDPs as a stand-alone treatment for prevention and treatment of biofilms not realistic. From a therapeutic point of view, activity of HDPs against existing (preformed) biofilms might be the most interesting feature. Many of the problems facing the use of HDPs as anti-biofilm agents are similar to their development as a new antibacterial agent against planktonic bacteria. These include high cost of production, short half-life in biological environments and their inhibition by serum or other biological components. On this basis it is, with the exception of topical applications, hard to envision HDP as a stand-alone treatment since a considerable local concentration of peptides at the biofilm surface is required. Combination with existing antibiotics is an interesting alternative to obtain possible synergistic effects lowering the required dose of peptide. Synergy has indeed been observed *in vitro*^7,30,37,38^. Stabilization of peptides using D-amino peptides, cyclization or other ways of protection of peptides, are other potential routes to increase activity. Interestingly, a complicating, but potentially beneficial factor in interpretation of HDPs activity of biofilms *in vivo* is that many HDPs are strong immune-modulatory molecules. In fact, immunomodulation often considered the main function of HDPs *in vivo*. This dual activity of HDPs could actually, again, be the basis for interesting combinational strategies in which one HDP such as CRAMP (strongest eradicator in our hands), can (partially) remove the biofilm, and a second HDP such as LL-37 or cCATH-2 (both strong immune-modulators) could stimulate the innate immune system to work on the remaining damaged biofilm.

## Materials and Methods

### Bacteria, media, and peptides

All biofilm experiments were performed using *Pseudomona aeruginosa* ATCC 27853 *(P*. *aeruginosa)*; Mueller-Hinton broth (MHB; Becton Dickinson Difco, Sparks, USA) was used to grow *P*. *aeruginosa* and formation of biofilm, and tryptic soy agar (TSA, Oxoid Ltd., Hampshire, UK) was used for colony counting; Thirteen HDPs (cCATH-1, 2 and 3, LL-37, K9CATH, CRAMP, PR-39, PMAP-36, PMAP- 23, eCATH1, 2 and 3 and IDR-1018) were synthesized using Fmoc solid-phase synthesis CPC scientific, Sunnycale, CA). All peptides were purified to a minimum purity of 95% by reverse phase high-performance liquid chromatography prior to biological testing. The amino acid sequences of peptides used in this study are shown in Table 1. Cy5-labeled peptide was produced by adding the fluorescent dye to the N-terminus through an Aminohexonic acid linker.

**Table 1:** Host Defence peptides used in this study.

Name	Sequences	Length	Origin
cCATH-1	RVKRVWPLVIRTVIAGYNLYRAIKKK	26	chicken
cCATH-2	RFGRFLRKIRRFRPKVTITIQGSARF	26	chicken
cCATH-3	RVKRFWPLVPVAINTVAAGINLYKAIRRK	29	chicken
LL-37	LLGDFFRKSKEKIGKEFKRIVQRIKDFLRNLVPRTES	37	human
CRAMP	GLLRKGGEKIGEKLKKIGQKIKNFFQKLVPQPEQ	34	mouse
K9	RLKELITTGGQKIGEKIRRIGQRIKDFFKNLQPREEKS	38	dog
PR-39	RRRPRPPYLPRPRPPPFFPPRLPPRIPPGFPPRFPPRFP	39	porcine
PMAP-36	GRFRRLRKKTRKRLKKIGKVLKWIPPIVGSIPLGCG	36	porcine
PMAP-23	RIIDLLWRVRRPQKPKFVTVWVR	23	porcine
eCATH1	KRFGRLAKSFLRMRILLPRRKILLAS	26	equine
eCATH2	KRRHWFPLSFQEFLEQLRRFRDQLPFP	27	equine
eCATH3	KRFHSVGSLIQRHQQMIRDKSEATRHGIRIITRPKLLLAS	40	equine
IDR1018	VRLIVAVRIWRR	12	designed

### Determination of MIC and MBC

Antimicrobial activity of HDP’s against planktonic *P*. *aeruginosa* was assessed using broth dilution assays. An overnight culture of *P*. *aeruginosa* was grown in MHB at 37 °C to mid-logarithmic phase and diluted to 2 × 10^6^ CFU/ml. In a round bottom polypropylene 96 wells plate 50 μl bacteria were mixed with 50 μl of HDP (final concentration 0–40 μM). MIC was defined as the concentration of peptide which inhibited visual growth of bacteria in the wells after 24 h incubation at 37 °C. Subsequently, wells without visual growth were plated out on TSA plates and incubated at 37 °C for 24 h to determine the MBC.

### Biofilm formation

An overnight culture of *P*. *aeruginosa* was cultured in MHB at 37 °C, and then diluted to 1 × 10^8^ CFU/mL in fresh MHB. Hundred μl of bacterial suspension, containing 0–20 μM of HDP, was added to each well of 96-well (flat bottom) polystyrene microtiter plates (Costar® 3595; Corning Incorporated). After the incubation, the medium was aspirated gently and the wells of the plates were washed three times with 150 μl phosphate buffered saline (PBS) to remove unattached bacteria.

For determination of the biofilm biomass, crystal violet (CV) staining was used. Hundred μl of 99% methanol was added per well for 10 min for fixation, then aspirated, and plates were allowed to dry. Wells were stained with 100 μl 0.04% CV (Klinipath, Duiven, The Netherlands) for 20 min. Excess colorant was eliminated by three successive washes with sterile PBS. Finally, bound CV was solubilized with 33% acetic acid and optical absorbance was determined at 630 nm, using a microtiter plate reader (FLUO star Omega, BMG LABTECH, Germany). In order to determine the number of viable bacteria, biofilms were solubilized with 200 μl 0.1% Triton X-100 (Serva, Heidelberg, Germany). Bacterial cells were serially diluted and spread on agar plates and incubated o/n at 37 °C and subsequently counted.

### Biofilm attachment and formation assay

In order to evaluate the effect of selected Host Defence Peptides (cCATH-1, cCATH-2, cCATH-3, LL-37, CRAMP and IDR1018) on the first phase of biofilm formation (attachment), 1 × 10^8^ CFU/mL *P*. *aeruginosa* was added to 96 wells plates in the presence of 0–40 μM of HDPs. After 30 min, 5 μl of the medium was diluted and spread on TSA plates to determine viability of planktonic bacteria in the medium. Subsequently, incubation was continued for 4 h after which biofilm mass and viability of bacteria in the biofilm was determined as described above. For the effect of HDPs on biofilm formation, biofilms were formed similarly as described above but further incubated until 24 h in the presence of the HDPs. After that, biofilm mass and viability was assessed.

### Effect of HDPs on preformed biofilms

Biofilms were prepared for 24 h in a 96-well tissue culture microtiter plate as described in 2.2, and washed three times with 150 μl PBS. Peptides in 50% MHB (0–20 μM) were added to every well, and the plates were incubated for 1 h at 37 °C. After incubation, biofilm mass and viability of bacteria inside the biofilm were assessed as described.

### Confocal laser scanning microscopy (CLSM)

An overnight growth culture of *P*. *aeruginosa* in MHB was adjusted to a final density of 1 × 10^8^ CFU/ml. Five hundred microliters of the bacterial suspension was added to an 8-well chambered coverglass (1.5 Borosilicate glass, Lab-Tek II chambered coverglass, Rochester, NY). The chambers were incubated at 37 °C on an orbital shaker (120 rpm). The medium was changed after 24 h to remove non-attached bacteria. After a total incubation of 48 h, biofilms were exposed to HDPs (20 μM) for 1 h at 37 °C. Biofilms were gently washed with 0.85% (wt/vol) NaCl and stained for 20 min in the dark at RT with the FilmTracer™ LIVE/DEAD^®^ Biofilm Viability Kit (Life Technologies Europe BV, Bleiswijk, The Netherlands) (final concentrations: 5 μM SYTO9 and 30 μM propidium iodide (PI)). After rinsing, biofilms were examined using confocal laser scanning microscopy on a Leica TCS SPE-II, Mannheim, Germany) using a 20x or 63x objective. Laser excitation was performed sequentially at 488(SYTO9) and 561 nm (PI) and emission was collected from 510 to 550 nm (SYTO9) and from 630 to 700 nm (PI). Image stacks were collected with a step size of 2.1μm or 0.46 μm (for 20x and 63x objective, respectively) at 600 Hz and analyzed with the Imaris 8.2 software (Bitplane, Zurich, Switzerland). In addition, in order to investigate the potential of cCATH-2 to penetrate into the biofilm, the fluorescently labeled homologue CY5-cCATH-2 was used. Upon treatment biofilms were stained as described, except that PI was not added to the solution and instead labelled peptide was detected using excitation/ emission of 635/670–700 nm for CY5-cCATH-2.

### Statistical analysis

All experiments were performed in at least three independent assays. Statistical analysis was performed by One-way analysis of variance using the post-hoc Dunnett analysis. Significant differences were defined as p < 0.05.

### Data availability

All data is available within the article and its supplementary information files, and from the corresponding author on reasonable request.

## Electronic supplementary material

Supplementary Material
