# Supplementary material for: Inhibition and Eradication of Pseudomonas aeruginosa Biofilms by Host Defence Peptides
Source: Sci Rep. 2018 Jul 11;8:10446. doi: 10.1038/s41598-018-28842-8 (PMC6041282; doi:10.1038/s41598-018-28842-8)
Supplement: Supplementary file 1 — Supplementary Material [file 41598_2018_28842_MOESM1_ESM.pdf]

Supplemental material to:

Inhibition and Eradication of *Pseudomonas aeruginosa* Biofilms by Host Defence Peptides

Hongwei Chen[1,2], Richard W. Wubbolts[3], Henk P. Haagsman[2], Edwin J.A. Veldhuizen[2*]

[1] College of Animal Science, Rongchang Campus, Southwest University, Chongqing 402460, China

[2] Department of Infectious Diseases & Immunology, Faculty of Veterinary Medicine, Utrecht University, Utrecht, The Netherlands.

[3] Department of Biochemistry and Cell Biology, Faculty of Veterinary Medicine, Utrecht University, Utrecht, The Netherlands.

* corresponding author: e.j.a.veldhuizen@uu.nl

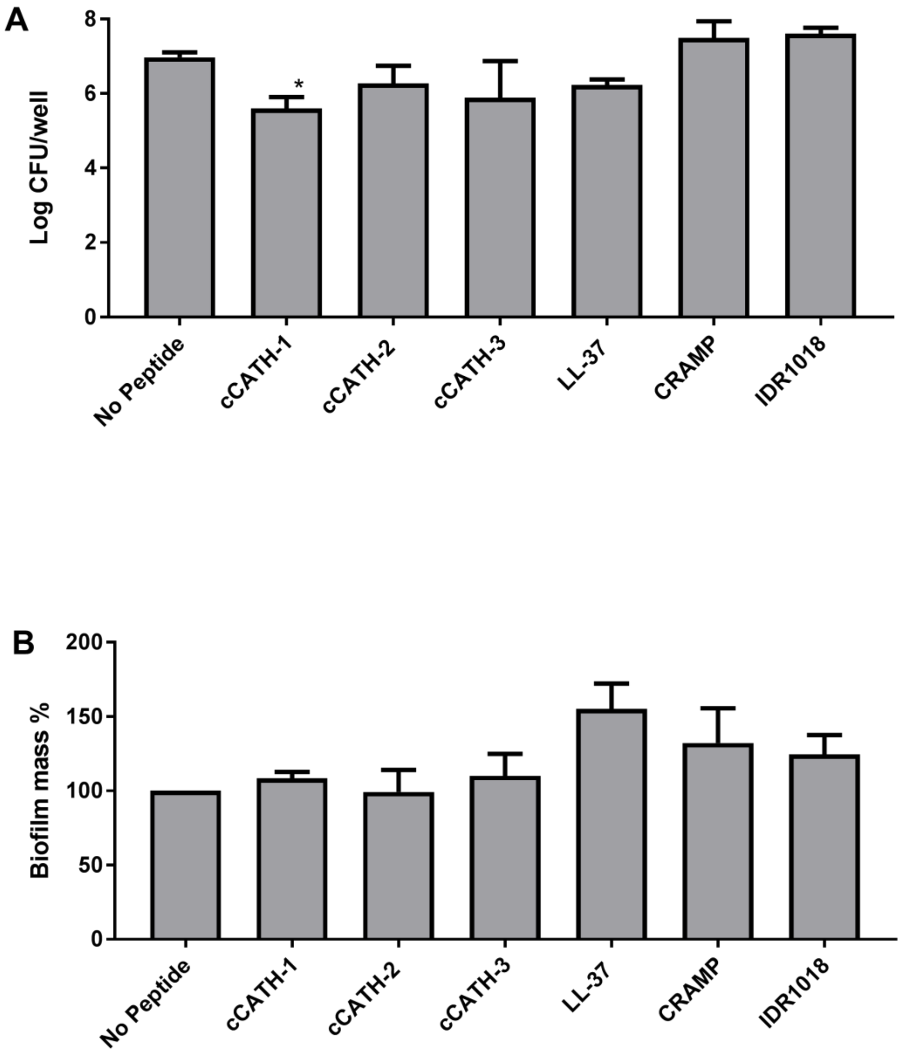

Figure S1. Effect of HDPs on biofilm formation.
P. aeruginosa biofilms were formed for 24 h at 37 °C in wells containing 0-20 μM cCATH-1,2 and 3, LL-37, CRAMP or IDR1018. Bars represent biofilm mass as determined by CV, while symbols and lines indicate the number of viable bacteria in the biofilm. Shown are averages of at least 3 independent experiments ± s.e.m. * indicates statistical significance compared to the non-peptide control.

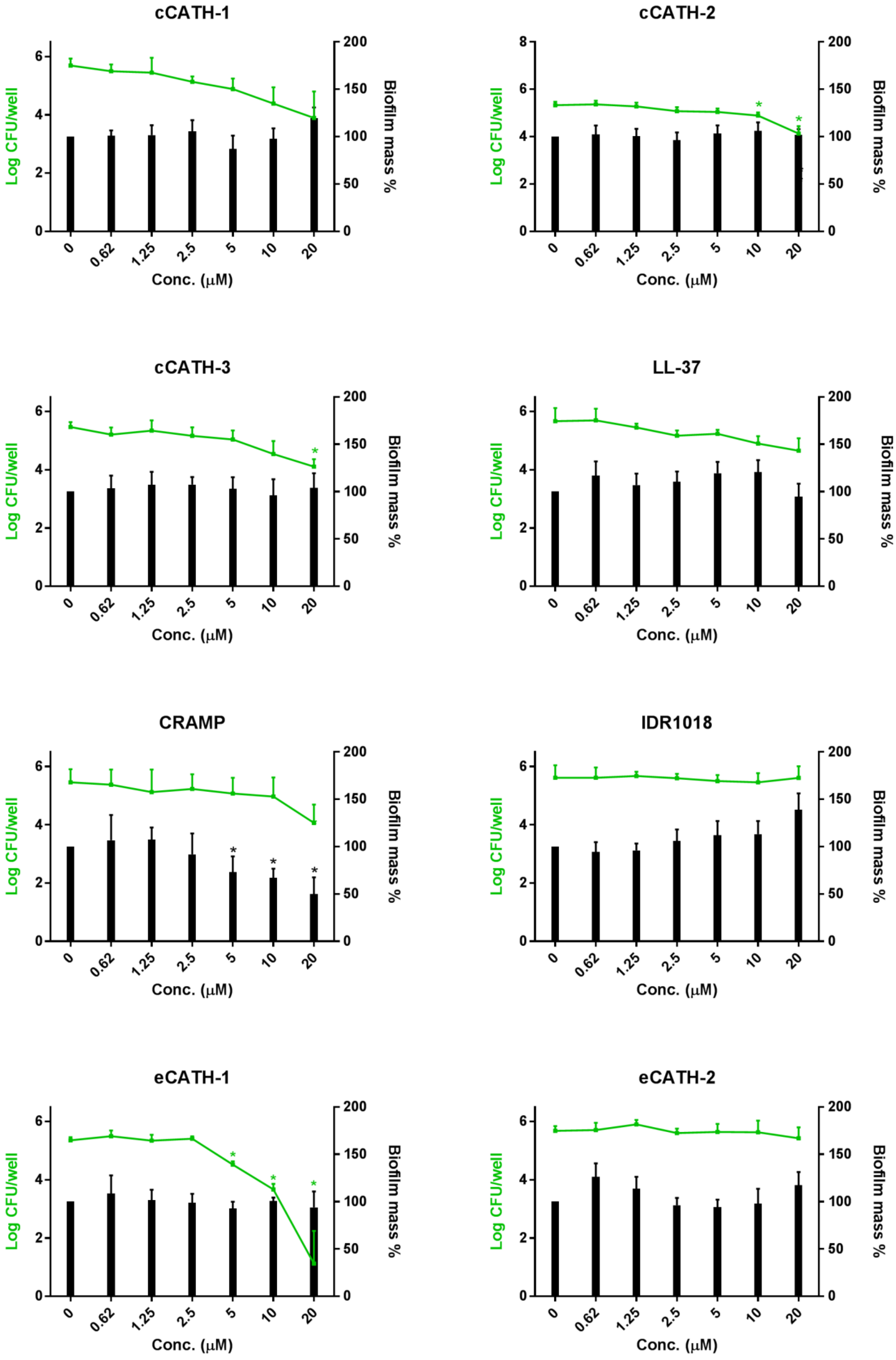
cCATH-1
cCATH-2
cCATH-3
LL-37
CRAMP
IDR1018
eCATH-1
eCATH-2
Log CFU/well
Biofilm mass %
Conc. (μM)
0
0.62
1.25
2.5
5
10
20
0
2
4
6
8
50
100
150
200
*

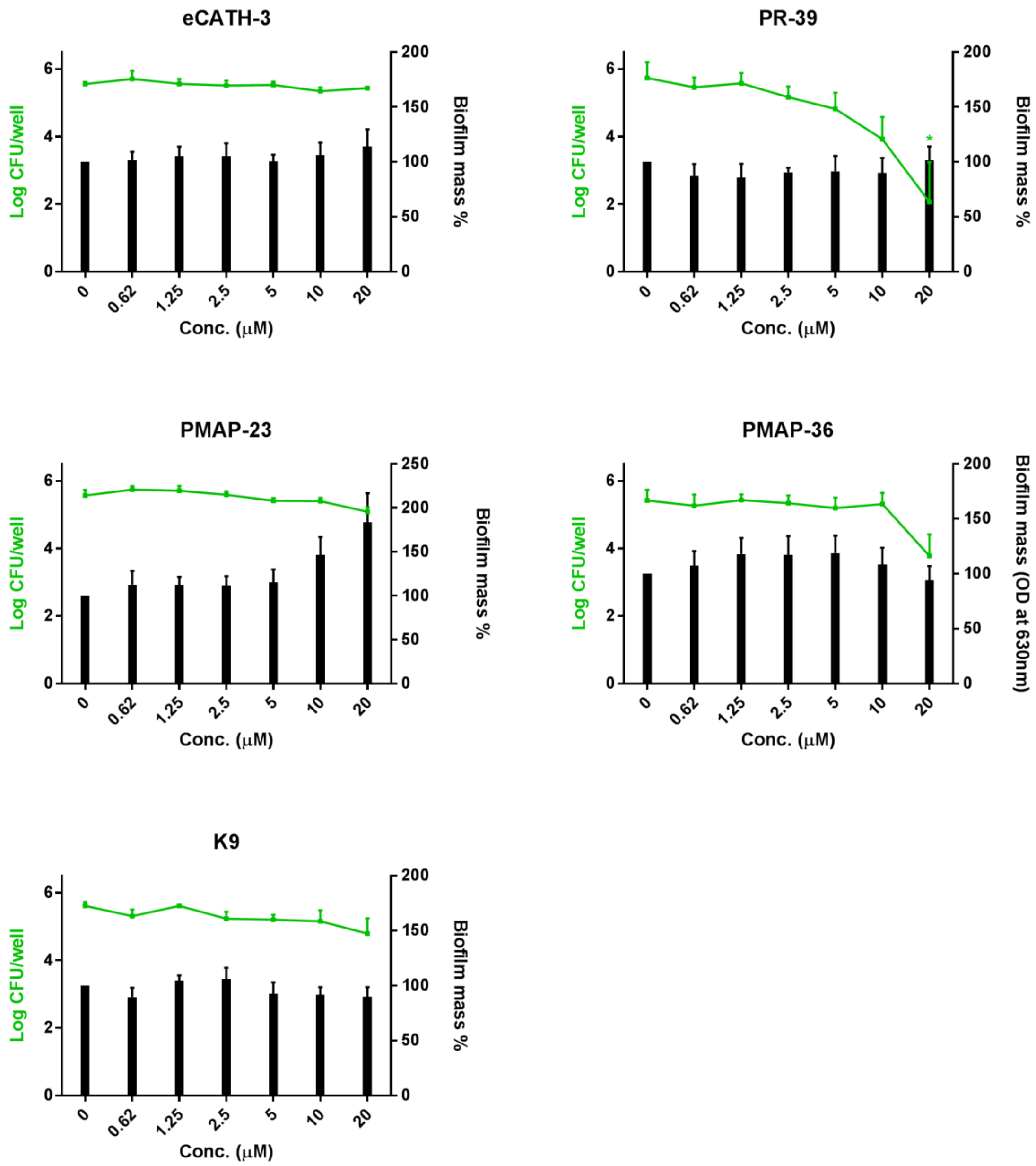

Figure S2. Activity of HDPs against preformed *P. aeruginosa* biofilm.
P. aeruginosa biofilms were formed for 24 h at 37 °C, and subsequently treated with the indicated peptides at 0- 20 μM for 1h. Viability and biofilm mass were determined using colony counting after solubilization of the biofilm, and crystal violet staining respectively. Bars represent biofilm mass, while symbols and lines indicate the number of viable bacteria in the biofilm. Shown are averages of at least 3 independent experiments ± s.e.m. *P<0.05,0.01,0.001 or 0.0001 vs control.

Table S1. Effect of cCATH-2 on preformed biofilms

[cCATH-2]	Viability (log CFU)<br>1h	2h	4h	6h	8h	24h
0	5.3 ± 0.1	6.1 ± 0.4	6.1 ± 0.1	6.8 ± 0.1	6.8 ± 0.1	7.0 ± 0.2
5 µM	5.1 ± 0.2	5.5 ± 0.2	5.8 ± 0.1	6.4 ± 0.4	6.7 ± 0.2	6.9 ± 0.1
10 µM	4.9 ± 0.1	5.5 ± 0.2	5.8 ± 0.2	6.8 ± 0.2	6.9 ± 0.1	6.8 ± 0.2
20 µM	4.1 ± 0.3	5.1 ± 0.1	5.7 ± 0.3	6.6 ±0.1	7.0 ± 0.3	7.0 ± 0.2
	Biofilm Mass (%)					
0	100	100	100	100	100	100
5 µM	103.2 ± 8.7	98.2 ± 14.9	126.3 ± 13.6	128.3 ± 12.7	140.1 ±13.1	84.4 ± 9.3
10 µM	106.0 ± 9.0	114.3 ± 14.1	119.6 ± 12.0	133.0 ± 11.6	169.9 ± 6.3	89.6 ±7.5
20 µM	101.8 ± 6.2	123.4 ± 11.3	119.4 ± 13.5	137.7 ± 17.0	145.1 ±19.6	100.8 ± 9.6

P. aeruginosa biofilms were grown for 24h and subsequently treated with 0, 5 , 10 or 20 µM cCATH-2. After different time points the number of viable bacteria in the biofilm and the biofilm mass were determined using colony count and crystal violet, respectively.

Table S2. MIC and MBC of HDPs against *P. aeruginosa*

HDP	MIC (µM)	MBC (µM)
cCATH-1	5-10	10-20
cCATH-2	5-10	10-20
cCATH-3	2,5-5	5-10
LL-37	10	10-20
CRAMP	40	>40
IDR1018	20-40	40
PMAP-23	40	>40
PMAP-36	5-10	10
PR-39	5-10	10
K9CATH	40	>40
eCATH-1	2.5-5	5-10
eCATH-2	>40	>40
eCATH-3	>40	>40

Antimicrobial activity of HDP's against planktonic *P. aeruginosa* was assessed using broth dilution assays. In a round bottom polypropylene 96 wells plate 50 µl 10^6 CFU/ml bacteria were mixed with 50 µl of HDP (final concentration 0-40 µM). MIC was defined as the concentration of peptide which inhibited visual growth of bacteria in the wells after 24 h incubation at 37 °C . Subsequently, wells without visual growth were plated out on TSA plates and incubated at 37 °C for 24 h to determine the MBC.
